# The development of a structured rating schedule (the BAS) to assess skills in breaking bad news

**DOI:** 10.1038/sj.bjc.6690423

**Published:** 1999-05

**Authors:** S J Miller, T Hope, D C Talbot

**Affiliations:** Wellcome Research Registrar, University of Oxford, Department of Psychiatry, Headington, Oxford, OX3 7JX, UK; Ethox, Division of Public Health and Primary Care, University of Oxford Institute of Health Sciences, Headington, Oxford, OX3 7LF, UK; University of Oxford, ICRF Medical Oncology Unit, The Churchill Hospital, Oxford, Headington, OX3 7LJ, UK

**Keywords:** breaking bad news, communication skills, assessment, cancer

## Abstract

There has been considerable interest in how doctors break bad news, with calls from within the profession and from patients for doctors to improve their communication skills. In order to aid clinical training and assessment of the skills used in breaking bad news there is a need for a reliable, practical and valid, structured rating schedule. Such a rating schedule was compiled from agreed criteria in the literature. Video-taped recordings of simulated consultations breaking bad news were independently assessed by three raters using the schedule and compared to three experts who gave global ratings. The primary outcome measures were internal consistency of the schedule and level of agreement between raters. The internal consistency was high with a Cronbach's alpha of 0.93. Agreement between raters using the schedule was moderate to good. The majority of the variation in scores was due to the differences in skills demonstrated in the interviews. The agreement between raters not using the schedule was poor. The BAS provides a simple to use, reliable, and consistent rating schedule for assessing skills used in breaking bad news. It could be a valuable aid to teaching this difficult task. © 1999 Cancer Research Campaign


British Journal of Cancer (1999) 80(5/6), 792–800
© 1999 Cancer Research Campaign
Article no. bjoc.1998.0423

### The development of a structured rating schedule (the BAS) to assess skills in breaking bad news

**SJ Miller[1], T Hope[2] and DC Talbot[3]**

[1]Wellcome Research Registrar, University of Oxford, Department of Psychiatry, The Warneford Hospital, Headington, Oxford OX3 7JX, UK; [2]Ethox, Division of Public Health and Primary Care, University of Oxford Institute of Health Sciences, Old Road, Headington, Oxford OX3 7LF, UK; [3]University of Oxford, ICRF Medical Oncology Unit. The Churchill Hospital, Headington, Oxford OX3 7LJ, UK

**Summary** There has been considerable interest in how doctors break bad news, with calls from within the profession and from patients for doctors to improve their communication skills. In order to aid clinical training and assessment of the skills used in breaking bad news there is a need for a reliable, practical and valid, structured rating schedule. Such a rating schedule was compiled from agreed criteria in the literature. Video-taped recordings of simulated consultations breaking bad news were independently assessed by three raters using the schedule and compared to three experts who gave global ratings. The primary outcome measures were internal consistency of the schedule and level of agreement between raters. The internal consistency was high with a Cronbach's alpha of 0.93. Agreement between raters using the schedule was moderate to good. The majority of the variation in scores was due to the differences in skills demonstrated in the interviews. The agreement between raters not using the schedule was poor. The BAS provides a simple to use, reliable, and consistent rating schedule for assessing skills used in breaking bad news. It could be a valuable aid to teaching this difficult task.

**Keywords:** breaking bad news; communication skills; assessment; cancer

Most doctors break bad news to patients as part of their clinical practice. This task is a frequent one for those involved in cancer care. How bad news is broken is important to cancer patients (Sell et al, 1993; Butow et al, 1996; Loge et al, 1997). There is a wealth of published advice on how to break bad news with several areas of agreement on how to do it effectively (see Ptacek and Eberhardt (1996) for a detailed review of published opinion).

Current opinion on how to break bad news suggests the use of a patient-led agenda (Fallowfield, 1993; Buckman and Kasson, 1992; Maguire and Faulkner, 1988). This is an interactive technique which involves first finding out what the patient currently knows, and then allowing the patient to control both the rate and amount of information delivery. The use of open questions and providing encouragement to the patient to ask questions are strategies that have been advocated to facilitate this process. Of key importance is the detection of important psychosocial factors both before and after the news is given such that the patient is encouraged to disclose feelings and concerns which are then explored by the health care professional.

There is empirical evidence that such disclosure and exploration reduces patient anxiety during the consultation (Macleod, 1991); lessens the risk for subsequent anxiety and depression (Harrison et al, 1994); and enables health care professionals to help the patient resolve his concerns which also lessens the risk of developing an affective disorder (Parle et al, 1996). Patients who ask questions and have them answered also demonstrate better adjustment (Butow et al, 1995). Coping theory (Lazarus, 1993) has been offered as an explanation of these phenomena (Parle et al, 1996, Ptacek and Eberhardt, 1996). In this model, coping is mediated through the individual patient's cognitive appraisal of the severity of the bad news and his personal resources to cope with it. The use of the techniques outlined above make patient appraisals amenable to influence by the doctor, enabling him to positively influence patient coping outcomes.

Despite the acknowledged importance of this patient-centred approach, breaking bad news remains difficult in clinical practice, and several recent observational studies have highlighted deficiencies in doctors' skills in this area. Ford et al (1996) demonstrated low levels of patient centredness and psychological focus amongst their sample of British oncologists. Patients asked few questions and 60% of all utterances came from the doctor. Four and a half times more biomedical than psychosocial information was exchanged in the first consultation. Clinicians relied on the use of closed questions and where psychosocial issues were disclosed by patients these were not followed up in the majority of cases. In a British surgical oncology setting, Burton and Parker (1997) found that when disclosing malignancy few surgeons enquired into any psychosocial aspects of the patient's experience. Eggly et al (1997), studying US junior doctors (residents), concluded that they were sub-standard in those elements that measured patient centredness. In addition to these observational studies, surveys of both senior and junior doctors have demonstrated a subjective awareness of the problem. Girgis et al (1997) surveyed a sample of Australian surgeons of whom 80% indicated a need for formal training and 13% identifying themselves as lacking in competence in breaking bad news. In a large UK postal survey, Gillard et al (1993) found that 54% of houseofficers (interns) felt that they had not received adequate training on breaking bad news.

*Received 28 May 1998*
*Revised 27 October 1998*
*Accepted 26 November 1998*

*Correspondence to:* SJ Miller, University of Manchester. CRC Psychological Medicine Group, Christie Hospital, Manchester M20 4BX, UK

All doctors have been urged to improve their communication skills (Stewart, 1995; Fallowfield, 1995; G. MC, 1993). A recent report from the Royal College of Physicians indicates that poor communication was the main cause of complaints about doctors (Royal College of Physicians, 1997). There is current interest in improving the communication skills of both under- and post-graduate doctors, since doctors who have not undergone specific training on how to give information are poor at doing so (Maguire et al, 1986*a*). However, inconsistencies may arise in teaching because, without structured guidance, experienced clinicians are unreliable when assessing clinical skills (Noel et al, 1992). Inter-rater reliability has been highlighted as particularly poor in the assessment of interviewing skills (Kalet et al, 1992), students have been scored on the basis of their liability rather than by specific behavioural skills they demonstrate. As a consequence, the ability of assessors to provide specific feedback on behaviour may be limited. The problem has been highlighted by Garg and colleagues (Garg et al, 1997) who have developed an educational programme for medical students specifically aimed at teaching how to break bad news. Although this course has been well-received by the participants over a 10-year period, the authors have not been able to draw any conclusions about the students' performance due to the lack of an adequate and validated method of assessment.

The aim of the research described in this paper was to develop a standardized way of assessing the communication skills necessary to break bad news effectively. There are three main purposes for such an assessment method: first, to assess the overall competence of individuals; second, to identify areas where skills are deficient; and third, to evaluate the effectiveness of training. Such an assessment must be effective over a wide range of different clinical abilities and problems, and be simple and relatively brief. We present here the development of such an assessment tool: the Breaking bad news Assessment Schedule (BAS, Appendix 1).

### MATERIALS AND METHODS

### Development of the rating scale

This was a two-stage process: first the content of the BAS was identified, and second the format of the BAS was decided.

In order to identify the key elements of the breaking bad news interview to be included in the rating scale, a literature search was undertaken using the MEDLINE and PSYCHLIT databases. Using the term 'bad news' as the descriptor, with 'English language articles' as a limiter, we searched databases back to 1966. A total of 281 references were initially identified. This number reduced to 79 when titles and abstracts were studied in order to select those relevant and to reject duplicate articles appearing on both databases.

Our intention was to identify the range of key behaviours which the authors of these works had suggested should be part of a bad news interview and which could be incorporated into an instrument designed to meet our aims. As noted by Ptacek and Eberhardt (1996), little of the published work is empirical, being mainly opinion and comment. Consequently, we did not weight authors, suggestions as to the frequency with which they appeared in the literature. We incorporated suggestions from the papers on three criteria: one, the practicality of being used in the assessment instrument; two, of having been demonstrated to be effective empirically (see Introduction); and three, when authors, suggestions disagreed with each other, we used our judgement both as teachers and clinicians experienced in breaking bad news as a final arbiter for inclusion. The behaviour of having a relative present during the consultation, one behaviour recommended by some authors, does not appear in the BAS (although identifying social support does) because we wanted to develop an instrument which could be used using just one simulated patient. An item addressing this could be added to the BAS to assess a setting where a relative or simulated relative was present. Through this process we identified 81 key behaviours which were consistent with each other and could be used in a single breaking bad news interview. For the purposes of scoring, these behaviours were grouped into 23 elements which appear as questions in the final BAS scale (see Appendix 1 for details).

Once the content of the BAS was identified it remained constant through each of the stages of development. At each stage of development the authors held a workshop where three videotapes were rated independently (see below) to assess the format of the BAS. On the basis of these workshops, the style of the BAS evolved through four versions before the final, version was agreed upon. It was this version that was subjected to the psychometric testing detailed below. The first version was based on an algorithm, but this proved too complex to be practical. The second was a checklist for the presence of the behaviours identified in the literature search. This also proved impractical, both as the list was too long and 'box ticking' focussed the rater on the minutiae of the consultation which detracted from the ability to rate the consultation in a relatively short time. The third and subsequent versions were of the format demonstrated in the Appendix. Alterations to the format were then made to subsequent versions with a view to both simplifying its use and increasing inter-rater agreement. These changes took the form of: clarifying the language to reduce ambiguity and increase specificity; switching to a scale with variable anchor points in order to reduce the likelihood of repeated response sets and make the rater read and think about the questions on an individual basis; introducing a five-point Lickert-type scale to allow a mid point to avoid raters being forced into a false dichotemization; and ensuring a variable number of specific behavioural points under each question to emphasize their nature for guidance. These changes were sufficient to ensure that no items were removed because of unreliability between the authors.

### Recruitment of subjects

Twenty-three health care professionals were recruited to simulate the role of the doctor (one nurse, one medical statistician, four medical students, seven senior house officers, four registrar and six consultants). They responded to either a letter or a poster approved by the Oxfordshire psychiatric research ethics committee. The purpose of a sample with such a range of clinical experience was to maximize the variety of the skills and experience in breaking bad news. This was required in order to ensure that the instrument would be effective over a wide range of abilities.

The use of simulated patients is well-established in teaching communication skills (Maguire et al 1986*b*; Cushing and Jones, 1995; Garg et al, 1997). Performance of interviewers with simulated patients is similar to that seen with real patients; the main advantage is that their use avoids exposing patients to the wide range of abilities needed to test the instrument. Furthermore, the content of interviews can be standardized, simulators can take part in a repeated number of interviews and interviews can be arranged

© Cancer Research Campaign 1999

**Table 1** Profiles of patient simulators

| Age | Sex | Diagnosis given by doctor |
|---|---|---|
| 65 | M | Acute myeloblastic leukaemia |
| 50 | F | Recurrent breast cancer |
| 42 | F | Breast cancer |
| 35 | F | Breast cancer |
| 35 | M | Recurrent testicular teratoma |

to suit educational or examination timetables. Posters asking for healthy volunteers were displayed in three Oxford teaching hospitals. Five subjects were recruited to simulate patients and selected to represent a range of ages and personal circumstances. These subjects were trained by the method previously described by Maguire et al (1986*b*) to play the role of a patient with a specific condition (Table 1). The scenarios were written to ensure a range of types of bad news, including diagnosis, prognosis, side-effects of treatment, disease progression and no effective treatment. This was required in order to ensure the instrument would be effective over a range of situations experienced by cancer patients. Following training, each simulated patient was interviewed by one of us (SM) using the techniques to elicit cognitive appraisal (outlined in the Introduction) specific to each individual simulator. These appraisals were then condensed into a ten-item checklist (see Appendix 2) which was used by raters to assess skills in eliciting concerns (question 16 in the BAS)

### Video-taped simulated breaking bad news scenarios

Each of the simulated doctors took part in one breaking bad news consultation with one of the simulated patients. The consultations were designed to mimic, as closely as possible, a real-life consultation in which the diagnosis of cancer or of recurrence was given to the patient. Consultations took place in a standardized room in which the simulated doctor was given the opportunity to rearrange the furniture. Five minutes were allowed to assimilate the relevant clinical information and a maximum of 15 min to conduct the consultation. After each interview, the participants were debriefed. Each of the simulated doctors was offered feedback on their performance. This took the form of an individual 1 h session watching the video with one of us (SM). The BAS was used to identify strengths and weaknesses of the performance and facilitate discussion. In turn, they gave feedback on the helpfulness of the BAS.

Twenty-three tapes were made in total, these were used for both the development of the rating scale and in the reliability studies.

### Inter-rater reliability

For a rating scale to have a high degree of utility it should not require a prior degree of expert knowledge for its reliable use. Thus, for the purposes of evaluating the BAS, three health care professionals, none of whom had any experience in teaching communication skills, acted as raters (a registrar in oncology, a registrar in psychiatry; and an oncology clinical nurse specialist). They were given training in the use of the BAS. This consisted of two sessions, a total of 3 h, rating three videotaped interviews using the BAS. After each video, the individual item scores were compared and discussed with one of us (SM) to clarify the rating for problematic questions. Portions of the tape were replayed and re-rated again, following discussion, to ensure uniformity between raters scores. After this training, the three raters independently rated the remaining 20 videotaped consultations in random order. These ratings were completed within 5 min of watching each taped recording. Inter-rater reliability was examined by comparison of these ratings.

### Comparison ratings

Three people experienced in teaching communication skills (two Fellows of the Royal College of Physicians, and a senior lecturer in medical ethics) independently rated the same 20 videotapes used in the inter-rater reliability study. They were informed that the videotapes were of a simulated interview in which bad news was broken and asked to carefully watch the tape and rate the performance of the simulated doctor in breaking bad news and conducting the interview. Each assessor was asked to give the simulated doctor a score between zero and 100 and to provide detailed written comments on the strengths and weaknesses of the consultation (these data were collected for purposes other than the aim of this paper and will be presented at a later date). These raters knew nothing of the content or structure of the BAS.

### Statistical analyses

The statistical analyses were carried out using the Statistical Package for Social Sciences (SPSS) version 7.5. In order for the BAS to be used for purposes of assessing and examining students and doctors in the skills used in breaking bad news an overall score needs to be generated. The BAS asks a series of questions the answers from which can then be combined to give a single numerical value. For this to be valid, it is important that all questions measure the same thing. The questions should be consistent and scores for each question should correlate with each other. Internal consistency of the BAS was measured using Cronbach's alpha statistic (Bland and Altman, 1997). This is a summary statistic which measures the overall correlation between the individual answers to questions and the total scores, with a value of 1 representing perfect correlation and O none.

Inter-rater reliability of the two groups of assessors was examined in two ways. First, the interviews were ranked by total scores and divided into quartiles. This was intended to mirror a situation where an assessment is used to divide skills levels into four categories (for example: outstanding, pass, borderline and fail), such as might be used in an examination. The weighted $\kappa$ statistic (Brennan and Silman, 1992) was used to calculate the level of agreement between raters using these four categories. This is a simple measure of the extent to which agreement between raters is better than might be expected by chance, with a value of 0 representing only chance agreement and 1 perfect agreement. The weighted $\kappa$ statistic does not provide data on the source of any disagreement.

Second, in order to investigate the sources of disagreement, the data were treated as continuous and an analysis on total scores was carried out. This took the form of an analysis of variance (ANOVA). This was used to calculate the Intraclass Correlation Coefficient (ICC), systematic bias, and random error, the principles of which are outlined below and described by Brennan et al (1992). Variation in the total scores is made up from two sources: first, there is variation due to the quality of the interviews or the

 © Cancer Research Campaign 1999

performance of the interviewer (which is equivalent to agreement amongst raters and is measured as the ICC); second, there is variation (disagreement) between the scores different raters give for the same interviews. Comparison between raters using a perfect scale would yield an ICC of 100%, i.e. complete agreement. Where disagreement between raters occurs it can be further divided into two independent sources: systematic bias and random error. Systematic bias occurs when individual raters consistently record scores higher or lower relative to other assessors, the so-called 'Hawk and Dove' effect. A scale with narrowly defined objective criteria for measurement will allow less scope for systematic bias. Random error, as its name suggests, follows no pattern, either between or within individual raters. For any method of measurement to be valid, random error should account for only a small proportion of the variation.

### RESULTS

### The BAS

The final version of the BAS is shown as Appendix 1. The 81 desirable behaviours identified in the literature search were grouped together as guidance points to facilitate the answering of 23 questions. These questions represent key elements that could be used in an effective patient-centred consultation in which bad news was broken. They are grouped together into five sections that appear in the chronological order one would expect to see in an actual interview: setting the scene; breaking the news; eliciting concerns; information giving; and general considerations. This facilitates using the BAS whilst watching an interview. The content of the BAS is arranged so that each of the biological, psychological, and social aspects of the encounter is assessed in an integrated manner.

### Ease of use of the BAS

Each of the three raters gave positive feedback overall on the BAS. They felt the language was clear, the Lickert-type scale was straightforward to use and the behavioural points under each question were helpful. Infrequently, raters experienced difficulty deciding between which number to give on individual questions. This was not associated with any particular questions or interviews. All were able to complete rating of the videotapes within 5 min of the time it took to watch them.

Twenty-two of the 23 simulated doctors took up the offer of a feedback session on their performance. The BAS proved useful in identifying specific strengths and weaknesses of individual components of the interview. It received favourable comments from the subjects that were similar to those given by the raters.

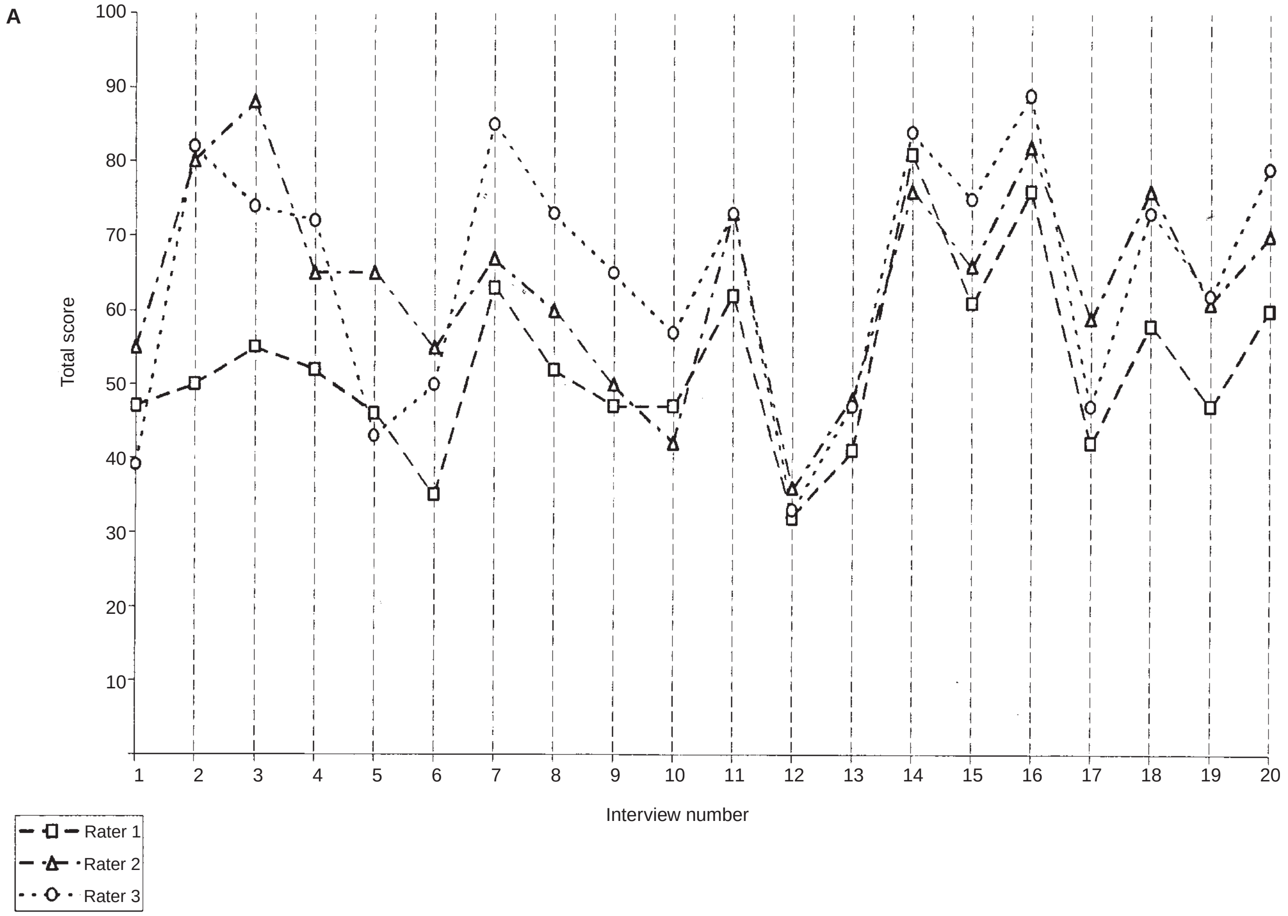

**Figure 1A** Total Scores for BAS Raters

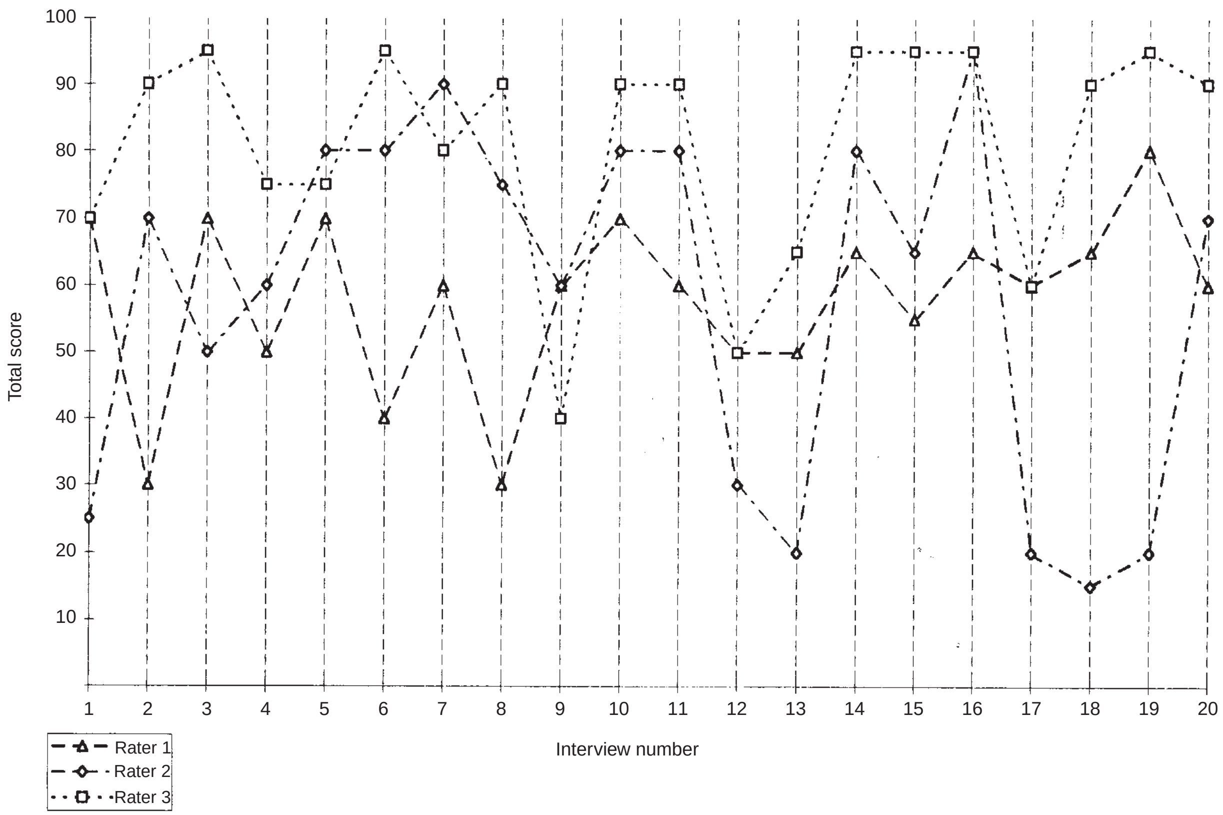

**Figure 1B** Total Scores for Non BAS Assessors

### Internal consistency

The Cronbach's alpha score for the BAS was 0.93.

### Inter-rater reliability

The total scores for each of the raters for all of the 20 videotaped consultations are shown in Figure 1A for those using the BAS and in Figure 1B for comparison raters. From comparison of these graphs it can be seen that scores given to the same interviews by raters using the BAS appear to agree more closely than those raters who did not use the BAS. For total scores, which have been ranked and divided into quartiles, the actual level of agreement between both set of raters as calculated using the weighted kappa statistic is shown in Table 2. This shows that, for raters using the BAS, agreement was moderate to good and considerably greater than that

**Table 2** The level of agreement of scores by quartiles (weighted κ values[a])

| | | | |
|---|---|---|---|
| **For raters using the BAS** | | | |
| | Raters 1 & 2 | Raters 1 & 3 | Raters 2 & 3 |
| | 0.4510 | 0.6817 | 0.6114 |
| For comparison raters | | | |
| | Raters A & B | Raters A & C | Raters B & C |
| | –0.0841 | 0.1904 | 0.1826 |

[a] Suggested interpretation of values: < 0.2 = poor, 0.21–0.40 = fair, 0.41–0.60 = moderate, 0.61–0.80 = good, 0.81–1.00 = very good.

**Table 3** The proportion of variation due to interviews, systematic bias and random error

| Variation | Raters using the BAS (%) | Comparison raters (%) |
|---|---|---|
| Due to Interviews (ICC) | 62 | 7 |
| Due to systematic bias | 17 | 29 |
| Due to random error | 21 | 64 |

 *© Cancer Research Campaign 1999*

which would be expected by chance whereas for comparison raters agreement was poor and little or nothing above that which would be expected by chance.

The values for the ICC, systematic bias and random error, calculated from the ANOVA, are given in Table 3. Both groups of raters showed bias, although this accounted for less of the variance for the raters who used the BAS. Random error is seen to account for 64% of the amount of variance in the scores given by the raters not using the BAS, three times that for the BAS raters. This higher level of bias, and to a greater extent random error, lead to a low ICC for the non-BAS group, such that only 7% of the variation in scores given for the interviews is accounted for by variation of the content of the interviews. Sixty-two per cent of the variance seen in the scores given for the interviews by raters using the BAS was accounted for by variation in the content of the interviews. For both sets of raters the ICC values approximate to the values of categorical agreement calculated by the weighted $\kappa$. This is what would be expected if the assumption that the data could be treated as continuous was correct. This justifies the use of the ICC as a measure of agreement for these data.

### DISCUSSION

In this paper we report the development of a structured method (the BAS) for rating professional skills at breaking bad news. The scale has proved easy to use and quick to apply to a videotape of such a consultation. The BAS has been used in two ways. First, to generate scores for individual components of the interview. This proved to be a useful tool in identifying strengths and weaknesses when we used it to give feedback on individual performances with simulated patients. Second, to provide a standardized score for the overall performance of an individual in a breaking bad news consultation. We used the BAS to evaluate the overall performance of a number of health care professionals simulating an interview in which bad news was given about cancer. The scale demonstrated a high degree of utility in that raters required minimum training and were able to rate whilst watching a 15-min consultation completing the task within 5 min of its ending. Inter-rater reliability was found to be moderate to good. Although some of the variation was due to rater bias, and random error, most of the difference, as one would hope, was accounted for by differences in skills demonstrated by the interviewers.

There are two further issues for scales of this kind. First, are they valid? Second, do they have an advantage over an unstructured global rating? The content of the scale, based upon areas of general agreement found in the literature, ensured a high degree of content validity. External validity, however, is difficult to measure because of the lack of a ‘gold standard’. One approach to a ‘gold standard’ would be patients’ own views on how they experience consultations. Building on the work presented here, a version of the BAS has been developed for patient use and is currently undergoing evaluation. A second approach to a ‘gold standard’ is to compare the BAS scores to the global ratings of experts. This approach is feasible if there is good agreement between the experts when rating interviews. However, the most prominent finding concerning the experts’ assessments in this study was their lack of agreement. The ANOVA showed that there was very little contribution to the variance from the quality of the interviews themselves. Bias contributed almost a third of the variation, and well over half was due to random error. Thus, the assessments made by the experts in our study were unreliable. Although our sample was small it concurs with data from other studies involving assessment of consulting skills (Kalet et al, 1992; Noel et al, 1992). It seems reasonable to conclude that unstructured global assessments are unreliable.

Breaking bad news is not a unitary construct. In the setting of cancer, bad news includes diverse communications with the patient. Initially, this may focus on the diagnosis and prognosis. Later, other issues such as disease progression, the effectiveness of treatment and the possibility of further treatment may predominate Older and more infirm patients may want less information (Casileth et al, 1980; Butow et al, 1996, 1997; Hamajima et al, 1996). Clearly, those giving bad news must adopt a flexible approach based on each individual’s wishes and needs. The BAS has been designed to accommodate such an approach whilst avoiding the reliability problems of global assessments. Its performance seems good over five different clinical scenarios that might occur in an oncology clinic. We believe our data suggest the BAS is ready for evaluation in real clinical situations. This could be done by evaluating videotapes or observing actual consultations directly. A further version of the BAS that uses audiotapes has now been developed and is currently undergoing evaluation.

Several authors in the field argue the importance of the doctor’s ability to handle the patient’s emotions as a key skill (Ptacek and Eberhardt, 1996). We agree that acknowledging and exploring the patient’s emotional state is important. In the BAS we have integrated these factors with biological and social considerations within several questions (1, 3, 10, 7, 18 and 21). This approach might not address the issue sufficiently directly and a future modification of the BAS to include specific questions wholly focused on emotional issues might be of value.

For many teaching programmes, the value of the BAS may be in identifying the specific strengths and weaknesses of the health professional or student to focus effective teaching. However, two further intended uses of this scale are to identify students, or qualified professionals, who had not reached a required standard in breaking bad news; and to evaluate the effectiveness of training programmes. For these purposes summing the individual element scores to generate an overall scores may help to identify relative overall competence. This approach would not be valid unless the scores from individual elements were consistent with each other. As the BAS has demonstrated the level of consistency (Cronbach’s $\alpha$ = 0.93) necessary for scales used to make clinical decisions (Bland and Altman, 1997). We believe the use of an overall score provides an effective basis for evaluating overall competence.

In conclusion, the BAS provides a reliable structured method for assessing skills in breaking bad news. It has three advantages over unstructured assessment by experts: first, it is much more reliable; second; it provides information on the different components of the breaking bad news consultation – potentially enabling strengths and weaknesses to be identified; and third, it is easy to teach to individuals who do not have any specific expertise. Further work is needed in establishing which components of the breaking bad news consultation are of importance to patients.

### ACKNOWLEDGEMENTS

The authors would like to thank all those that took part in the study as raters, simulated doctors and simulated patients; the ICRF Medical Oncology Unit, Oxford for providing facilities; and the Wellcome Trust for providing funding for SM as a Research Registrar. We gratefully acknowledge advice on overall design of

*© Cancer Research Campaign 1999*

the project from Professor David Sackett, comments on the third draft of the BAS from Angella Hall, statistical advice from Jenni Thompson and data management from Ruth Whitehouse.

### REFERENCES

Bland JM and Altman DG (1997) Statistics notes. Cronbachs alpha. *Br Med J* **314**: 572

Brennan P and Silman A (1992) Statistical methods for assessing observer variability in clinical measures. *Br Med J* **304**: 1491–1494

Brennan P, Silman A, Black C, Bernstien R, Coppock J, Maddison P, Sheeran T, Stevens C and Wollheim F (1992) Reliability of skin involvement measures in scleroderma. *Br J Rheumatol* **31**: 457–460

Buckman R and Kason Y (1992) *How to Break Bad News – a Protocol for Healthcare Professionals*. Toronto University Press: Toronto

Burton MV and Parker RW (1997) Psychological aspects of cancer surgery: surgeons' attitudes and opinions. *Psycho-oncology* **6**: 47–64

Butow PN, Dunn SM, Tattersall MHN and Jones QJ (1995) Computer-based interaction analysis of the cancer consultation. *Br J Cancer* **71**: 1115–1121.

Butow PN, Kazemi JN, Beeney LJ, Griffin A, Dunn SM and Tattersall MHN (1996) When the diagnosis is cancer, patient communication experiences and preferences. *Cancer* **77**: 2630–2637

Butow PN, Maclean M, Dunn SM, Tattersall MHN and Boyer MJ (1997) The dynamics of change: patients' preferences for information, involvement and support. *Ann Oncol* **8**: 857–863

Cassileth BR, Zupkis RV, Sutton Smith K and March V (1980) Information and participation preferences among cancer patients. *Annals of internal Medicine* **92**: 832–836

Cushing AM and Jones A (1995) Evaluation of a breaking bad news course for medical students. *Med Edu* **29**: 430–435

Eggly S, Afonso N, Rojas G, Baker M, Cardozo L and Robertson RS (1997) An assessment of Residents' competence in the delivery of bad news to patients. *Acad Med* **72**: 397–399.

Fallowfield LJ (1993) Giving sad and bad news. *Lancet* **341**: 476–478

Fallowfield LJ (1995) Psychosocial interventions in cancer. *Br Med J* **311**: 1316–1317.

Ford S, Fallowfield LJ and Lewis S (1996) Doctor–Patient interactions in oncology. *Soc Sci Med* **42**: 1511–1519.

Garg A, Buckman R and Kason Y (1997) Teaching medical students how to break bad news. *Can Med Assoc J* **156**: 1159–1164

Gillard JH, Dent THS, Aarons EJ, Crimlisk HL, Smyth-Piggot PJ and Nicholls MHN (1993) Preregistration houseofficers it the Thames regions: changes in quality of training after four years. *Br Med J* **307**: 1176–1179

Girgis A, Sanson-Fisher RW and McCarthy WH (1997) Communicating with patients: Surgeons' perceptions of their skills and need for training. *Aust NZ J Surg* **67**: 775–780

GMC (1993) *Tomorrow's Doctors*. General Medical Council: London

Hamajima N, Tajima K, Morshita M, Hyodo C, Sakakibara N, Kawai C and Moritaka S (1996) Patients' expectations of information provided at cancer hospitals in Japan. *Jpn-J-Clin-Oncol* **26**: 362–367

Harrison J, Maguire P, Ibbotsen T, Macleod R and Hopwood P (1994) Concerns, confiding and psychiatric disorder in newly diagnosed cancer patients: a descriptive study. *Psycho-oncology* **3**: 173–179

Kalet A, Earp J and Kowlowitz V (1992) How well do faculty evaluate the interviewing skills of medical students. *J Gen Intern Med* **7**: 499–505

Lazarus RS (1993) Coping theory and research: past, present and future. *Psychosom Med* **55**: 234–247

Loge JH, Kaasa S and Hytten K (1997) Disclosing the cancer diagnosis: the patients' experiences. *Eur J Cancer* **33(6)**: 878–82

Macleod R (1991) Patients with advanced breast cancer: the nature and disclosure of their concerns. MSc thesis, University of Manchester.

Maguire P and Faulkner A (1988) Communicate with cancer patients: 1. Handling bad news and difficult questions. *Br Med J* **297**: 907–909

Maguire P, Fairbairn S and Fletcher C (1986*a*) Consultation skills of young doctors: II Most young doctors are bad at giving information *Br Med J* **292**: 1576–1578

Maguire P, Fairbairn S and Fletcher C (1986*b*) Consultation skills of young doctors: 1. Benefits of feedback training in interviewing as students persist. *Br Med J* **292**: 1573–1576

Noel GL, Hebers JE, Caplow MP, Cooper GS, Pangaro LN and Harvey J (1992) How well do internal medicine faculty members evaluate the clinical skills of residents? *Ann Int Med* **117**: 757–765

Parle M, Jones B and Maguire P (1996) Maladaptive coping and effective disorders among cancer patients. *Psychol Med* **26**: 735–744

Ptacek JT and Eberhardt TL (1996) Breaking bad news. A review of the literature. *JAMA* **276**: 496–502

Royal College of Physicians (1997) College Report: Improving communication between doctors and patients. *J R Coll Phys Lond* **3**: 258–259

Sell L, Devlin B, Bourke SJ, Munro NC, Corris PA and Gibson GJ (1993) Communicating the diagnosis of lung cancer. *Respir Med* **87**: 61–63

Stewart MA (1995) Effective physician communication and health outcomes: a review. *Can Med Assoc J* **152**: 1423–1433

### APPENDIX 1

### Breaking bad news Assessment Schedule (BAS)

When marking please place a circle round the number which reflects the score you wish to give. The points below each question are for guidance only. When the doctor has delivered the diagnosis stop the tape and mark the first two sections before restarting and continuing to mark.

***A. Setting the scene:*** this section looks at whether the doctor facilitated an initial rapport *before breaking the bad news*. This can be done by providing an environment which allows private and comfortable communication, by the doctor introducing him/herself, and by the doctor showing an interest in the patient as an individual.

1. Did the doctor arrange the environment?

very well 5 _ 4 _ 3 _ 2 _ 1 poorly

The doctor may have

- placed the chairs at an angle which allowed unforced eye contact?
- ensured that the desk was not in-between him/her and the patient?
- ensured that the wastepaper basket was out of the way?
- prepared for the patient becoming upset, for example by placing the tissues so the patient could reach them?
- taken measures to prevent interruptions, for example unplugging the telephone?

2. Did the doctor use an appropriate greeting and introduction?

definitely 5 _ 4 _ 3 _ 2 _ 1 not at all

The doctor may have

- stood up to greet the patient?
- established the patient's name?
- introduced him/herself using his/her own name?
- given a brief description of his/her occupation?
- shown the patient where to sit?

3. Did the doctor show interest in the patient's current state of well-being and personal circumstances at the beginning of the interview?

definitely 5 _ 4 _ 3 _ 2 _ 1 not at all

The doctor may have

- used open questions?
- established recent events for the patient?
- established the patient's physical state?
- asked how the patient felt emotionally?
- enquired into the patients social circumstances?
- given the patient time to finish their statements?

 © Cancer Research Campaign 1999

***B. Breaking the news:*** this section specifically focuses on whether the doctor was sensitive to this patient's perspective when he/she delivered the news (the establishment of rapport is scored in the above section). The amount of information to give each individual patient may vary depending on what the patient already knows. Individual patients may vary in the amount of information they wish to receive during this interview, and in the rate at which they assimilate the news.

4. **Before** breaking the news did the doctor check what this patient knew already?

carefully 5 _ 4 _ 3 _ 2 _ 1 not at all

Did the doctor
- ask the patient what he/she believed was the nature of their problem?
- enquire into what the patient thought the purpose of this meeting was?
- check if the patient had thoughts about the possible outcomes from this consultation?
- ensure that he/she understood the patient's perspective at this stage of the interview?

5. **Before** breaking the news did the doctor introduce it with sensitivity?

definitely 5 _ 4 _ 3 _ 2 _ 1 not at all

Did the doctor
- gently alert the patient to the fact that what followed was going to be important, before using any specific terms?
- take the lead from the patient as to whether to speak or listen after introducing the news?

6. When delivering the news did the doctor allow the patient to decide the detail and language used?

definitely 5 _ 4 _ 3 _ 2 _ 1 not at all

Did the doctor
- begin by using non-specific lay terminology?
- respond to the patients cues, or ask the patient if he or she wanted more detail, before becoming more specific?
- check that the patient was satisfied with his/her own understanding of the terms used?

7. Did the doctor allow the patient to set the pace for the delivery of the news?

definitely 5 _ 4 _ 3 _ 2 _ 1 not at all

Did the doctor
- deliver appropriate information when it was asked for?
- give the news at a rate which gave the patient time to think and respond?
- check that the patient had understood and assimilated what had been said before giving more information?

8. Did the doctor use an appropriate pause after giving the news?

definitely 5 _ 4 _ 3 _ 2 _ 1 not at all

Did the doctor
- allow the news about the diagnosis and its implications to sink in?
- give the patient time to respond?
- appropriately break the silence if the pause was too long?

***C. Eliciting concerns:*** this section focuses on whether the doctor actively attempted to gain a clear idea of the personal implications and meaning of the news to this patient, and the concerns that it generated.

9. Did the doctor **specifically** invite questions?

definitely 5 _ 4 _ 3 _ 2 _ 1 not at all

The doctor may need to invite questions repeatedly.

10. Did the doctor **explicitly** attempt to obtain a complete list of the patient's concerns?

definitely 5 _ 4 _ 3 _ 2 _ 1 not at all

Did the doctor explore
- he patient's feelings and emotions about the news just given?
- the patient's concerns about treatment?
- the patient's concerns about prognosis?
- the concerns arising from family and relationship issues?
- the patient's concerns about the effect on their social setting, for example their employment?

11. Did the doctor **explicitly** check which areas were most important to the patient?

carefully 5 _ 4 _ 3 _ 2 _ 1 not at all

Did the doctor
- ask the patient which issues were important to talk about during this meeting?
- ask in which order the patient wanted to talk about these issues?

***D. Information giving:*** this section looks at aspects other than giving the news itself.

12. Did the doctor give information tailored to the patient's expressed concerns?

entirely 5 _ 4 _ 3 _ 2 _ 1 not at all

Did the doctor
- give information in a manner which related to the patient's expressed concerns?
- answer the patient's questions?

13. Did the doctor clearly explain any information given so that the patient understood?

definitely 5 _ 4 _ 3 _ 2 _ 1 not at all

Did the doctor
- give information in an ordered and logical manner?
- use terms appropriate to this patient using plain English and avoiding jargon?
- check that the patient understood, and offer clarification?
- summarize points for the patient?

14. Did the doctor manage to focus on any positive aspects?

definitely 5 _ 4 _ 3 _ 2 _ 1 not at all

Did the doctor
- frame treatment options in a positive way?
- achieve a good balance between explaining benefits and side-effects?

*© Cancer Research Campaign 1999*

- manage to give correct information about the prognosis without extinguishing hope?

15. Was the content of the interview factually accurate?

always 5 _ 4 _ 3 _ 2 _ 1 frequently inaccurate

- If all the information given was factually correct this should gain full marks.
- If the doctor admitted to uncertainty or lack of knowledge this should still allow full marks.
- Marks should be deducted for incorrect statements, undue optimism, premature reassurance, or unjustified negativity.

***E. General considerations***: the following points relate to the interview as a whole.

16. How many of the patient's concerns from the attached list* were **aired**?

All ten 5 _ 4 _ 3 _ 2 _ 1 none at all

- 1 to 3 concerns from the attached list = 1 mark
- 4 or 5 = 2 marks
- 6 or 7 = 3 marks
- 8 or 9 = 4 marks
- all 10 = 5 marks

17. How many of the key areas of the patient's concerns were **touched upon**?

all of them 5 _ 4 _ 3 _ 2 _ 1 none at all

Each of the following five key areas should be touched upon to obtain full marks

- treatment
- prognosis
- feelings and emotions
- family and relationship issues
- effect on social circumstances

*See Appendix 2 for a sample list

18. Were the psychosocial issues which the patient flagged up during the interview **explored**?

fully 5 _ 4 _ 3 _ 2 _ 1 not at all

Did the doctor

- acknowledge: the patient's feelings and emotions; and the effects on family and relationships, and social circumstances?
- allow the patient to talk about these issues?
- ask questions about them?
- enter into a dialogue?

19. Did the doctor manage to appear supportive during the interview?

always 5 _ 4 _ 3 _ 2 _ 1 not at all

Did the doctor

- show warmth?
- show emotional supportiveness?
- convey a sense that this really mattered to the doctor?
- convey a personal sense of strength and resourcefulness that was available to help the patient?

20. Did the doctor use appropriate body language during the interview?

definitely 5 _ 4 _ 3 _ 2 _ 1 not at all

Did the doctor

- maintain an appropriate level of eye contact?
- look interested and alert to the patients needs?
- show a competent and caring professional manner?

21. Did the doctor avoid appearing clumsy during the interview?

never clumsy 5 _ 4 _ 3 _ 2 _ 1 often clumsy

Did the doctor

- introduce difficult topics gently?
- deal with painful issues sensitively?
- show flexibility and sensitivity to the patient's needs?
- avoid non sequitur?
- avoid using phrases that were inappropriate?

22. Did the doctor tailor the pace of the interview to suit the patient?

definitely 5 _ 4 _ 3 _ 2 _ 1 not at all.

Did the doctor

- let the patient speak without interruption?
- respond to the patient's cues regarding timing and delivery?
- deliver appropriate information when it was asked for?
- use pauses where appropriate to give the patient time to think and respond?
- check that the patient had finished with a topic before moving on to another?

23. Did the doctor manage the time available?

very well 5 _ 4 _ 3 _ 2 _ 1 poorly

Did the doctor

- sensitively make the patient aware of how much time was available for discussion?
- mention the opportunity of further interviews to the patient?
- cover the important issues in this session?
- make a plan for future action?
- bring the interview to a conclusion?

### APPENDIX 2

### Sample concerns list for breast cancer patient

1. Is the diagnosis certain
2. Has the disease spread
3. How will treatment affect my prognosis
4. What will be the side-effects of treatment
5. Will I still be able to work during treatment
6. Will I still be able to do my hobbies
7. Will my sister get this too
8. Will this stop me finding a partner
9. Will I become infertile
10. How will telling my father affect him

*© Cancer Research Campaign 1999*

## References

[PDF_00548] Bland J. M., Altman D. G. (1997). Cronbach's alpha.. BMJ.

[PDF_00552] Brennan P., Silman A., Black C., Bernstein R., Coppock J., Maddison P., Sheeran T., Stevens C., Wollheim F. (1992). Reliability of skin involvement measures in scleroderma. The UK Scleroderma Study Group.. Br J Rheumatol.

[PDF_00550] Brennan P., Silman A. (1992). Statistical methods for assessing observer variability in clinical measures.. BMJ.

[PDF_00557] Burton M. V., Parker R. W. (1997). Psychological aspects of cancer surgery: surgeons' attitudes and opinions.. Psychooncology.

[PDF_00559] Butow P. N., Dunn S. M., Tattersall M. H., Jones Q. J. (1995). Computer-based interaction analysis of the cancer consultation.. Br J Cancer.

[PDF_00561] Butow P. N., Kazemi J. N., Beeney L. J., Griffin A. M., Dunn S. M., Tattersall M. H. (1996). When the diagnosis is cancer: patient communication experiences and preferences.. Cancer.

[PDF_00564] Butow P. N., Maclean M., Dunn S. M., Tattersall M. H., Boyer M. J. (1997). The dynamics of change: cancer patients' preferences for information, involvement and support.. Ann Oncol.

[PDF_00572] Eggly S., Afonso N., Rojas G., Baker M., Cardozo L., Robertson R. S. (1997). An assessment of residents' competence in the delivery of bad news to patients.. Acad Med.

[PDF_00575] Fallowfield L. (1993). Giving sad and bad news.. Lancet.

[PDF_00576] Fallowfield L. (1995). Psychosocial interventions in cancer.. BMJ.

[PDF_00578] Ford S., Fallowfield L., Lewis S. (1996). Doctor-patient interactions in oncology.. Soc Sci Med.

[PDF_00580] Garg A., Buckman R., Kason Y. (1997). Teaching medical students how to break bad news.. CMAJ.

[PDF_00582] Gillard J. H., Dent T. H., Aarons E. J., Crimlisk H. L., Smyth-Pigott P. J., Nicholls M. W. (1993). Preregistration house officers in the Thames regions: changes in quality of training after four years.. BMJ.

[PDF_00585] Girgis A., Sanson-Fisher R. W., McCarthy W. H. (1997). Communicating with patients: surgeons' perceptions of their skills and need for training.. Aust N Z J Surg.

[PDF_00589] Hamajima N., Tajima K., Morishita M., Hyodo C., Sakakibara N., Kawai C., Moritaka S. (1996). Patients' expectations of information provided at cancer hospitals in Japan.. Jpn J Clin Oncol.

[PDF_00595] Kalet A., Earp J. A., Kowlowitz V. (1992). How well do faculty evaluate the interviewing skills of medical students?. J Gen Intern Med.

[PDF_00597] Lazarus R. S. (1993). Coping theory and research: past, present, and future.. Psychosom Med.

[PDF_00599] Loge J. H., Kaasa S., Hytten K. (1997). Disclosing the cancer diagnosis: the patients' experiences.. Eur J Cancer.

[PDF_00607] Maguire P., Fairbairn S., Fletcher C. (1986). Consultation skills of young doctors: I--Benefits of feedback training in interviewing as students persist.. Br Med J (Clin Res Ed).

[PDF_00605] Maguire P., Fairbairn S., Fletcher C. (1986). Consultation skills of young doctors: II--Most young doctors are bad at giving information.. Br Med J (Clin Res Ed).

[PDF_00603] Maguire P., Faulkner A. (1988). Communicate with cancer patients: 1. Handling bad news and difficult questions.. BMJ.

[PDF_00610] Noel G. L., Herbers J. E., Caplow M. P., Cooper G. S., Pangaro L. N., Harvey J. (1992). How well do internal medicine faculty members evaluate the clinical skills of residents?. Ann Intern Med.

[PDF_00613] Parle M., Jones B., Maguire P. (1996). Maladaptive coping and affective disorders among cancer patients.. Psychol Med.

[PDF_00615] Ptacek J. T., Eberhardt T. L. (1996). Breaking bad news. A review of the literature.. JAMA.

[PDF_00619] Sell L., Devlin B., Bourke S. J., Munro N. C., Corris P. A., Gibson G. J. (1993). Communicating the diagnosis of lung cancer.. Respir Med.

[PDF_00621] Stewart M. A. (1995). Effective physician-patient communication and health outcomes: a review.. CMAJ.

